# Detection of the epidemic of the H3N8 subtype of the equine influenza virus in large-scale donkey farms

**DOI:** 10.1080/23144599.2020.1739844

**Published:** 2020-03-31

**Authors:** Yu Yongfeng, Sun Xiaobo, Xia Nan, Zhang Jingwen, Liu Wenqiang

**Affiliations:** College of Agriculture, Liaocheng University, Liaocheng, Shandong, China

**Keywords:** Large-scale donkey farm, EIV, H3N8 subtypes, antibody

## Abstract

To monitor the occurrence of equine influenza in large-scale donkey farms in Liaocheng City, Shandong Province, serological investigation and sequence analysis of HA/M protein gene of equine influenza virus (EIV) were carried out. Samples (n = 65) of the lung and nasal swab were collected in six different large-scale donkey farms and detected with RT-PCR for HA and M protein gene. The homology and evolution of HA and M genes were analysed with known sequences. Antibody titres of serum samples (n = 120, unvaccinated) level was determined by the HI test. The average seropositive rate was 32.5% (39/120) with great diversity among different populations. The positive rate of EIV HA/M protein gene was 21.5% (14/65) by RT-PCR. The equine influenza H3N8 virus was confirmed by gene sequencing, and the homology of the sequence was 99.77% with isolates from Northeast China (equine/heilongjiang/1/2010), consistent with the input of donkeys. This suggested that EIV has become an important threat to large-scale donkey farms in Liaocheng and threats from the input area must be vigilant.

## 1. Introduction

Equine influenza virus (EIV) is an acute and contagious infectious disease of horses, donkeys and other equine family caused by the equine influenza A virus of genus *Orthomyxovirus* [1]. Influenza A viruses are subtyped according to their surface glycoprotein haemagglutinin (HA) and neuramindase (NA). The HA mediates virus entry into the host cell by binding to the sialic acid receptors and mediating fusion of viral and host membranes [2]. There are two major subtypes, H7N7 and H3N8, which have been isolated from horses [3]. World Health Organization (OIE) regulates that horse flu is a legally reported animal epidemic. It is classified as the third category of animal epidemic disease in China [4]. The characteristic clinical symptoms of influenza virus infection in equine animals include high fever, cough, serous nasal juice and female abortion. If not treated in time, it can also lead to pneumonia, enteritis, emphysema and even death [5]. The epidemic of EIV is extremely strong, once infected, it will quickly spread to the whole population. The mode of transmission is mainly through direct contact or through people or other animals indirectly. Horse transport, especially the cross-border transport of horse races, is the main reason for the spread of horse flu from one country to another [6].

**Figure 1.**
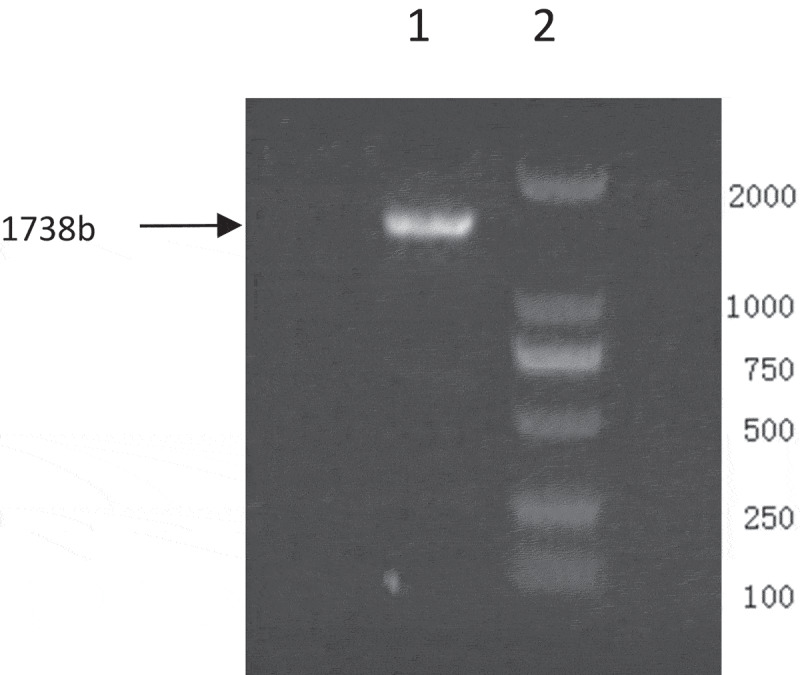
PCR products of HA gene. (1.HA gene 2.DL-2000 Marker)

**Figure 2.**
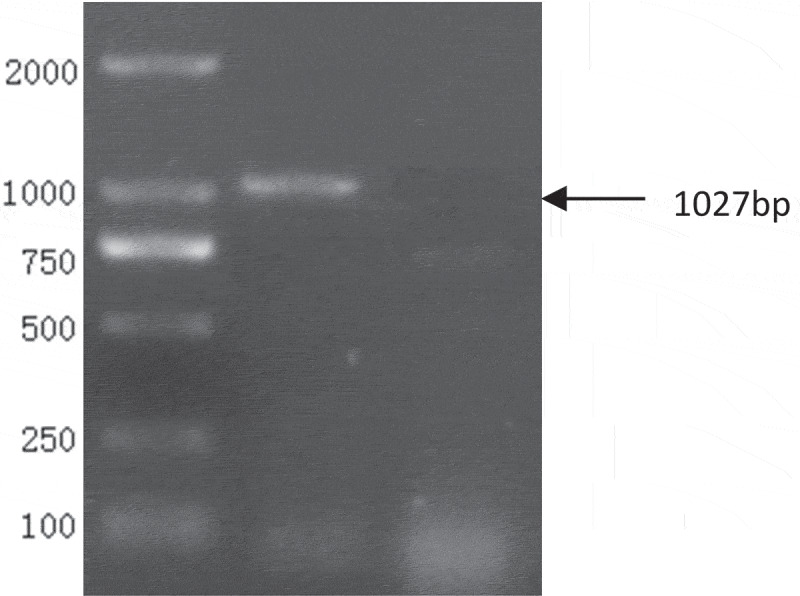
PCR products of M gene. (1.DL-2000 Marker 2.M gene)

Horse flu is very important infectious disease endangering donkey breeding and horse breeding. In recent years, it has caused varying degrees of economic losses in several countries around the world [7]. Horse flu was first found in Xinjiang in China, followed by outbreaks in Jilin, Heilongjiang and Xuzhou in 2005 [8].

Two hundred and seven large-scale donkey farms have been built around Liaocheng City, Shandong Province. With the increasing of stocking density and more frequent transport flows, the threat of epidemic viruses and infectious diseases can not be avoided or ignored. At present, donkeys raised in Liaocheng City are mainly used for food and the production of Ejiao. As there is no vaccine against EIV in China, the outbreak of influenza will inevitably involve the application of antibiotics, thus affecting the quality and medicinal value of Ejiao as a high-grade health product [9]. The aims of this work are to identify the EIV H3N8 subtype isolates in large-scale donkey farms and speculate on its possible source.

## 2. Materials and methods

### 2.1. Collection of samples

The principal materials tested in this work were nasal cotton swabs, lungs and serum from six independent farms in Liaocheng City (the stock ranges from 300 to 1000). Nasal cotton swabs were taken from adult donkeys with fever, runny nose and cough in a large-scale donkey farm around Liaocheng. The lungs are derived from dead donkeys. Serum is randomly drawn from the donkey herd. These animal experiments were approved by the Animal Welfare Committee of the local institution, and all procedures were carried out in accordance with the guidelines of the China Animal Protection Association.

### 2.2. Design and synthesis of primers

Specific primers for HA and M gene fragments according to the conservative sequence (GenBank-registered number JQ265982) was designed by Primer5.0 Primer Design Software and synthesized by Sangon Biotech. Upstream primers:(HA)5’-ATATTTCTGTCAATCATGAAGAC-3’ (M) 5’-AAGATGAGTCTTCTGACCGA-3’. Downstream primers: (HA) 5’-CTATCAGTTTACTCAAATGCAA-3’ (M) 5’-TTACTCCAGCTCTATGTTGAC-3’. The length of the target gene HA is1738bp and M is 1027 bp.

### 2.3. Hemagglutination inhibition (HI) test

Serum antibodies of each donkey farm were detected by inactivated antigen of EIV H3N8 subtype (NECVB company) and antibody titres were determined by HI test. Negative serum and pig serum were added as controls. (The test results are counted according to different regions and genders of donkeys.)

### 2.4. Reverse transcription and amplification

Main molecular reagents were purchased form Takara Company (China.) RNA samples isolated from above organs and tissues freshly reverse transcribed in 42°C for 1 h and termination reaction at 85°C 5 min (enzyme inactivation) using Oligo dT (0.5 µg/µl) 1 µl and AMV RT (10 U/µl). PCR was performed on an aliquot of the resulting cDNA template using Mastercycler Personnal (Eppendorf Co, Germany) as follows: pre-denaturation 95°C for 3 min to 95°C for 30 s, 53°C for 1 min, 72°C for 1 min, 34 cycles. Extension reaction 72°C 5 min. The PCR products were identified by 1% agarose gel electrophoresis.

### 2.5. Purification and sequencing of PCR products

After the target PCR product is detected by electrophoresis, one HA sample and one M sample were purified using a gel recovery kit according to the operating manual and sequenced by SANGON biotech by using upstream and downstream primers of HA and M gene.

### 2.6. Sequence analysis

The sequencing results were registered with NCBI and compared with the known virus genes by BLAST. The phylogenetic trees of the HA gene and M gene were constructed by MEGA software using the neighbour-joining approach. The bootstrap consensus tree was estimated from 1000 replicates.

## 3. Results

### 3.1. Antibody test results

According to the HI test, the positive rate of 120 serum samples from 6 farms ranged from 20% to 45%, with an average of 32.5% (Table 1.) The statistical results showed that the positive rate of antibody in six fields was higher, and the positive rate of female donkey was the highest in different populations, which was 45% (Table 2.)

**Table 1.** Detection results of antibody levels in different fields

Grouping	Detection quantity	Positive number	Positive rate	Antibody titer		
				≤1:2	1:2–1:8	1:16≥
1	20	7	35.0%	13	4	3
2	20	6	30.0%	14	3	3
3	20	6	30.0%	14	2	4
4	20	4	20.0%	16	3	1
5	20	7	35.0%	13	3	4
6	20	9	45.0%	11	5	4
Total	120	39	32.5%	13.50	3.33	3.16

Antibody titres are average.

**Table 2.** Detection results of antibody levels in different populations

Group Name	Detection quantity	Positive number	Positive rate	Antibody titer		
				≤1:2	1:2–1:8	1:16≥
Male	40	12	30.0%	28	6	6
Female	40	18	45.0%	22	8	10
Donkey foal	40	9	22.5%	31	6	3
Total	120	39	32.5%	27.00	6.67	6.33

Antibody titres are average.

### 3.2. RT-PCR amplification

The PCR products were identified by 1% agarose gel electrophoresis and photographed with gel imager (Figures 1 and 2). With the M/HA protein gene as the reference, the positive rate of RT-PCR was 21.5% (14/65).

After sequencing (Sangon biotech), the sequences have been deposited in the GenBank database under the accession MK880355 for HA gene and MK886767 for M gene.

### 3.3. Analysis of HA and M proetin sequence

**Figure 3.**
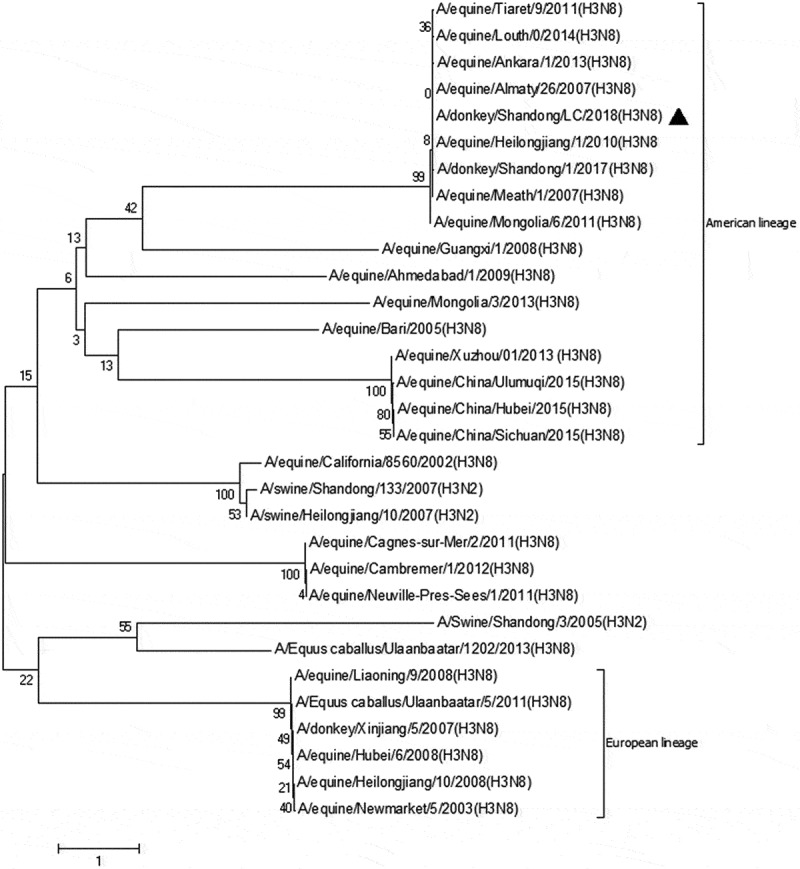
Genetic evolutionary tree analysis of HA gene

**Figure 4.**
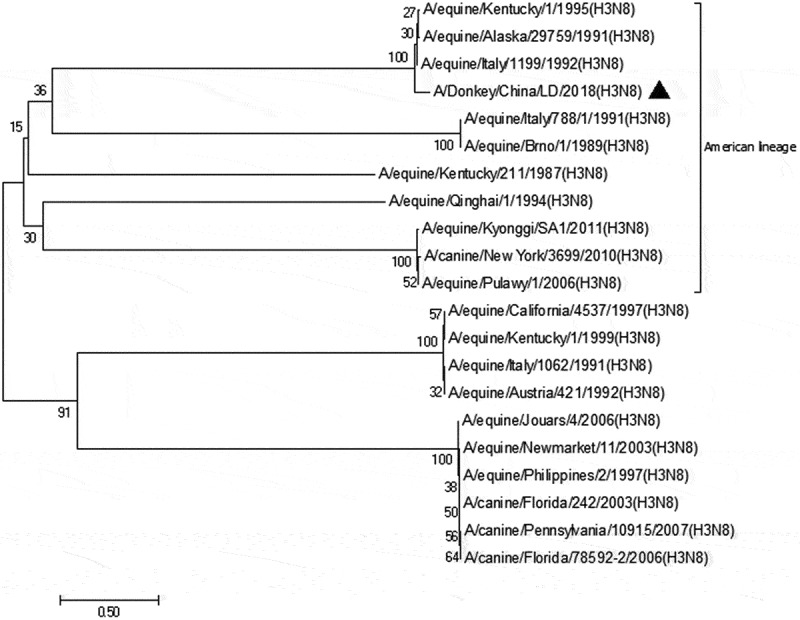
Genetic evolutionary tree analysis of M gene

The BLAST results and the phylogenetic tree indicated that HA gene sequence of the strain had the highest homology with equine/heilongjiang/1/2010 (registration number: JQ265982.2), which was 99.77% belonging to the same branch. Strains from Ankara, Mongulia, Louth (Irland) and Tiaret since 2011 also should be included in the same branch. This branch is dominated by strains from 2007 to 2008 in northern China with 99.24%~99.29% homology. It was significantly further from the other branch, which contained isolated strains of equine from California (USA), Guangxi (China) and Ahmedabad (India) with 98.52%~98.22% homology (Figure 3.) Sequence of M proteins had the highest homology with equine/ltaly/1199/1992 (registration number: CY032342.1) and have a high homology with other strains around the world (Figure 4.)

## 4. Discussion

Since 2015, hundreds of large-scale donkey farms have been built in Liaocheng City, Shandong Province, and then extended to Hebei, Shanxi Province and other regions, which are completely different from traditional free culture. Original sporadic distribution of EIV is also likely to be popular.

In the late 1980 s, EIV diverged into two antigenically distinct lineages [10], American and Eurasian, and since then, the American lineage has further diverged into the Kentucky, South American and Florida sub‐lineage clades I and II [11]. In recent years, there have been continuous reports of equine influenza outbreaks in China but the relevant gene sequences were not updated. Most of the EIV isolated are belonging to Florida Type II H3N8 [12]. It has high homology with a Mongolia/1/2008 strain isolated from Mongolia and A/equine/Jammu-Katra/6/00 isolated from India. However, the strain isolated from Japan belongs to a different evolutionary branch in the genetic evolutionary tree [13]. At the same time, the EIV isolated in China in the 1990 s is also in a unique evolutionary branch [14]. Key amino acids at several antigen sites of the HA protein of EIVs that cause an epidemic in China have undergone significant changes compared with other branch-chain EIV [15]. It is obvious that the HA sequence of the donkey source isolates belongs to a large branch in northern China, while the M sequence belongs to the kentucky branch of the phylogenetic tree. This complex distribution also confused about whether new mutations in donkeys happened needing a further research.

In this study, 21.5% positive rate of the samples associated with the M protein gene with respiratory diseases shows that it is necessary to further strengthen the surveillance of equine influenza epidemic risks. Antigenic and genetic information suggest that the evolution of EIV is mainly based on the mutations found on the HA surface glycoprotein [16]. In the study, it was found that the strain had the highest homology with the EIV in Heilongjiang region, which was speculated to be due to the epidemic in Liaocheng area caused by the introduction of donkey population from northeast China through the comparison of the HA sequence of the strain with other known sequences.

Since all of our samples were from non-immunized donkey farms, 32.5% positive rate of EIV, suggesting that they had been exposed and infected with EIV, which deserves vigilance. Whether this happened in donkey’s origin, transportation or in Shandong Province is unknown. In any case, regular serological and molecular biological monitoring are necessary in these areas.

## 5. Conclusion

The research findings suggested that EIV had brought about a serious threat to large-scale donkey farms. Veterinarians must assess this threat and strengthen biosafety measures to prevent its epidemic, especially around Liaocheng in Shandong Province.

## References

[1] Jing H, Wei M, Jianhua S, et al Construction and expression of prokaryotic expression Vector of HA1 Gene of H3N8 subtype Equine Influenza virus [J]. J Xinjiang Agric Univ. 2013;36(1):12–15.

[2] Dimitrov D. Virus entry: molecular mechanisms and biomedical applications. Nature Rev Microbiol. 2004;2(2):109–122. 15043007 10.1038/nrmicro817 PMC7097642

[3] Scholtens G, Steele H, Dowdle R, et al U.S. Epizootic of Equine Influenza, 1963. Public Health Rep (1896–1970). 1964;79(5):393–402. PMC1915427 14153655

[4] Taozhen J, Yuehuan L, Jian L, Chunhua H, Jie P, Ping'an L, Haitao W, Junjie Z, Jingyi Z, Zhijun M, Dingren B Isolation and identification of H3N8 subtype equine influenza virus in North China in 2007 [J]. Chin J Vet Med. 2010; 30(5):607–608.

[5] Burashev Y, Strochkov V, Sultankulova K, et al Complete genome sequencing of two equine influenza A(H3N8) virus strains isolated in Kazakhstan.[J]. Genome Announc. 2018;6(26). DOI:10.1128/genomeA.00574-18. PMC6025920 29954896

[6] Feifei GE, Jian L, Houbin J, Dequan Y, Jinping Z Isolation and Identification of a H3N8 Subtype Equine Influenza Virus and Analysis of Whole Gene Evolution [J]. Chin J Vet Med. 2015; 35(9):1435–1440+1467.

[7] Zhicheng Z, Jiande S, Nan Y, et al Study on the risk of global horse influenza [J]. Anim Quarantine China. 2010;27(12):45–48.

[8] Qinghai L, Hongsheng S, Xiuhua. C Research status of equine influenza [J]. Anim Husbandry Vet Sci Technol Inf. 2005;21(6):19–21.

[9] Li. G Present situation and analysis of veterinary drug residues in livestock and poultry products [J]. Anim Husbandry Veterinary Sci Technol Inf. 2019;35(3):140.

[10] Feifei GE, Jian L, Houbin J, et al Isolation, identification and whole gene evolution analysis of a strain of H3N8 equine influenza virus [J]. Chin J Vet Med. 2015;35(9):1435–1440+1467.

[11] Worobey M, Han G, Rambaut A A synchronized global sweep of the internal genes of modern avian influenza virus. Nature. 2014;508(7495):254–257. 24531761 10.1038/nature13016 PMC4098125

[12] Gahan J, Garvey M, Asmah Abd Samad R Whole genome sequencing of the first H3N8 equine influenza virus identified in Malaysia.[J]. Pathogens. 2019;8(2). DOI:10.3390/pathogens8020071 PMC6630255 31083430

[13] Yang H, Xiao Y, Meng F, et al Emergence of H3N8 equine influenza virus in donkeys in China in 2017[J]. Vet Microbiol. 2018;214:1–6. 10.1016/j.vetmic.2017.11.033 29408020

[14] Zheng Q Isolation and identification of equine influenza virus and study on a virus replicon vector vaccine [D]. Harbin: Northeast Agricultural University; 2012;(9):89.

[15] Xiaolei W, Huijuan D, Young J, et al Identification and sequence analysis of HA gene of a H3N8 subtype equine influenza virus [J]. Adv Anim Med. 2018;39(4):19–23.

[16] Xinyu T, Lien SM, Keun NM, et al Isolation and characterization of equine influenza virus (H3N8) from an equine influenza outbreak in Malaysia in 2015.[J]. Transbound Emerg Dis. 2019. 10.1111/tbed.13218 PMC6852086 31059176

